# Structure of a DNA G-Quadruplex Related to Osteoporosis with a G-A Bulge Forming a *Pseudo-loop*

**DOI:** 10.3390/molecules25204867

**Published:** 2020-10-21

**Authors:** Martina Lenarčič Živković, Jan Rozman, Janez Plavec

**Affiliations:** 1 Slovenian NMR Centre, National Institute of Chemistry, Hajdrihova 19, 1000 Ljubljana, Slovenia; jan.rozman@gmail.com; 2 Central European Institute of Technology, Masaryk University, Kamenice 753/5, 62500 Brno, Czech Republic; 3 EN-FIST Centre of Excellence, Trg OF 13, 1000 Ljubljana, Slovenia; 4 Faculty of Chemistry and Chemical Technology, University of Ljubljana, Večna pot 113, 1000 Ljubljana, Slovenia

**Keywords:** G-quadruplex, NMR spectroscopy, structure, osteoporosis

## Abstract

Bone remodeling is a fine-tuned process principally regulated by a cascade triggered by interaction of receptor activator of NF-κB (RANK) and RANK ligand (RANKL). Excessive activity of the *RANKL* gene leads to increased bone resorption and can influence the incidence of osteoporosis. Although much has been learned about the intracellular signals activated by RANKL/RANK complex, significantly less is known about the molecular mechanisms of regulation of *RANKL* expression. Here, we report on the structure of an unprecedented DNA G-quadruplex, well-known secondary structure-mediated gene expression regulator, formed by a G-rich sequence found in the regulatory region of a *RANKL* gene. Solution-state NMR structural study reveals the formation of a three-layered parallel-type G-quadruplex characterized by an unique features, including a G-A bulge. Although a guanine within a G-tract occupies *syn* glycosidic conformation, bulge-forming residues arrange in a *pseudo-loop* conformation to facilitate partial 5/6-ring stacking, typical of G-quadruplex structures with parallel G-tracts orientation. Such distinctive structural features protruding from the core of the structure can represent a novel platform for design of highly specific ligands with anti-osteoporotic function. Additionally, our study suggests that the expression of *RANKL* gene may be regulated by putative folding of its G-rich region into non-B-DNA structure(s).

## 1. Introduction

DNA can adopt various structures outside the double stranded margins of classical Watson-Crick geometries, which can interactively regulate biological processes such as replication, transcription, translation, and splicing [1,2,3,4,5,6]. The spatiotemporal formation of particular biologically relevant secondary structure is directed by several prerequisites in terms of a specific cellular environment, including cation concentration, presence of different proteins, molecular crowding, variations in pH, and oxidative stress [1,7,8,9,10,11,12]. Additionally, DNA regions that form these secondary structures usually differ from the surrounding DNA by distinct sequential elements, such as palindromic sequences or sequences with uncommon nucleotide distribution [1,3,6,13,14]. DNA regions, rich in tracts of guanines intervened by short (usually 1–7 nucleotides long) random sequences, are prone to self-associate into four-stranded structures called G-quadruplexes [2,4,15]. G-rich sequences able to fold into G-quadruplex structures are not randomly dispersed throughout the genome, but enriched within regions associated with gene regulation, including promoters, introns and UTRs, and at the ends of the chromosomes, where they play an important role in telomere biology [13,14,16,17,18,19,20]. In specific localized environments the formation of non-canonical DNA structures can even be preferred over B DNA form [21].

The core of the G-quadruplex structure is composed of stacked G-quartets, Hoogsteen hydrogen-bonded planes of four guanines, which are connected by short random loop-forming sequences in different orientations. Due to various possible options of G-quartet geometries and several loop combinations, G-quadruplexes are considered to be a very polymorphic structural family [2,15,19,22,23,24]. Individual G-strand, one of the four pillars of the G-quadruplex scaffold, is usually established by sequentially successive guanines, which participate in the formation of consecutive G-quartets. Next to G-quadruplex cores from four continuous G-strands, several structures with interrupted arrangements of guanines in one of the G-strands have been reported [25,26,27,28,29,30]. These, for example, involve a *Pu24* structure from the human *c-myc* promoter, where a discontinuous G-strand is formed by a guanine from the 3′-end and two guanines from one of the middle G-tracts [25]. Similar structural element was observed in *c-kit87up* sequence from the human *c-kit* promoter, where two guanines from the 3′-G-tract connect into a discontinuous G-strand with an isolated non-G-tract guanine from the middle of the sequence [26]. Additionally, two adjacent guanines in a G-strand can be connected via bulge, which usually protrudes from the G-quadruplex core, while surrounding guanines connect into a discontinuous G-strand [27,28,29,30]. A systematic study showed that stable G-quadruplexes are able to form despite introduction of bulges that vary in size or number per structure [27]. However, all up-to-date determined high-resolution DNA G-quadruplex structures with bulges contain one single-nucleotide G-strand protrusion [27,28,29,30]. Although the same length, these bulges vary in residue type; described structures include thymine [27,28], cytosine [29], and adenine [30] bulges, while G-quadruplex structure bearing guanine bulge was not reported so far.

Pathogenic conditions that affect the human skeleton, such as osteoporosis, disrupt balanced bone remodeling by accelerating differentiation and maturation of osteoclasts. The key regulator of osteoclast’s activity is receptor activator of NF-κB (RANK) that interacts with RANK ligand (RANKL) formed by osteoblasts [31,32,33,34]. The interaction initiates a cascade of intracellular signaling events and promotes bone resorption that in the case of excessive activity leads to osteoporosis [31,32,33,34]. Usually, the treatment of osteoporosis includes application of denosumab, a fully human monoclonal antibody to RANKL, which blocks its binding to RANK. This inhibits the development and activity of osteoclasts, which leads to decreased bone resorption and increased bone density [35,36,37]. Interfering with *RANKL* activity plays an important role in combat against osteoporosis. Thus, next to direct inhibition of RANKL/RANK interaction, influencing the *RANKL* expression could represent a promising way in a treatment of osteoporosis. One of the recently introduced ways to reduce the amount of RANKL protein is siRNA-mediated knockdown. Injections of RANKL siRNA-loaded virus-like-particles (VLPs) demonstrated a successful decrease in RANKL mRNA and protein levels in rats [38,39]. A 35% knockdown of membranous RANKL in osteoporosis-affected bones was achieved. Although this did not result in significant improvements of morphological or biomechanical properties in the early stage of osteoporosis, one has to take into consideration that the method is still under development.

Additionally, gene expression can be notably influenced by the formation of G-quadruplexes in the regulatory regions of a gene [13,14,16,17,18]. Recently, we assessed folding of a 20-nt G-rich sequence from the regulatory region of a *RANKL* gene (designated RANwt), characterized by three GGG tracts and one G-tract with four guanines [40]. RANwt in the presence of KCl folds into structurally polymorphic sample, supposedly due to four successive guanines in the first G-tract of the sequence. Introduction of two simple individual modifications, where edge guanines G1 and G4 from the GGGG tract were substituted with thymines, led to samples with single, although different, G-quadruplex folds. In our recent study we described a two-quartet G-quadruplex structure stabilized by A•G•A and G•G•G base triads, which forms upon G4-to-T4 modification of RANwt (referred to as RAN4) [40]. Unprecedented fold uncovered in our structural study was the first to imply that formation of non-canonical structures may play role(s) in the disease mechanism of osteoporosis by regulating the expression of *RANKL* gene.

The ability of RANwt to form other G-quadruplexes, different from RAN4, indicates possible topology-dependent regulation of *RANKL* gene expression. It has been shown before that different G-quadruplex structures can affect the gene transcription differently, revealing the importance of individual conformation for function [41]. Since switching between different G-quadruplex structures may be slow with respect to processes in a cell cycle, the formation of non-canonical structure(s) right after the double-stranded DNA unwinding might be the most significant on biologically relevant time scale(s) and can be as such used as an attractive target for gene expression inhibition [41,42]. Therefore, we deemed it essential to gain insights into the structural details of a G-quadruplex that can, next to RAN4, be formed by a modified RANwt sequence. In this work, we focus on a sequence with G1-to-T1 modification (RAN1), which predominantly forms a parallel-stranded three-layered G-quadruplex. Since the presence of unfolded oligonucleotide in solution impeded further structural characterization, additional modification of loop-forming A17 into thymine was introduced. The high-resolution structure with double (G1T, A17T) modification, herein referred as RAN1*, revealed that one of the guanines from the 3′-edge of the third G-tract together with successive adenine forms a G-A bulge. It appears that in order to facilitate base stacking with different G-quartets, bulge residues G14 and A15 occupy a *pseudo-loop* arrangement, while adjacent guanine G16 resides in *syn* glycosidic conformation. RAN1* structure highlights the potential role of non-G-tract guanines on G-quadruplex folding, structure, and, furthermore, function. At the same time, it offers novel insights into exciting structural elements that can be used for the development of highly selective ligands that may serve as an innovative approach in osteoporosis treatment in the future.

## 2. Results

### 2.1. Favoring a Single Conformation by Sequence Modifications of RANwt

**Figure 1:**
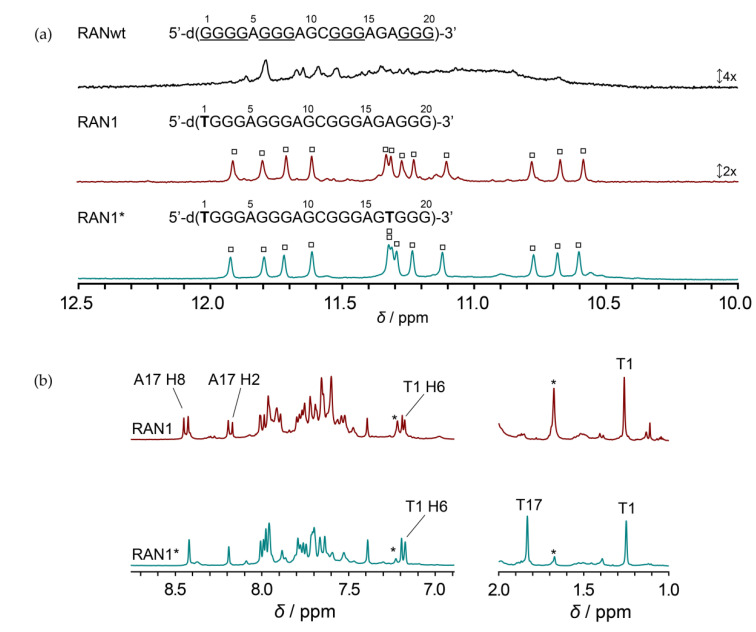
Comparison of 1D ^1^H NMR spectra. (**a**) Imino regions of ^1^H NMR spectra of wild-type (RANwt) and modified (RAN1 and RAN1*) G-rich sequences from the regulatory region of the *RANKL* gene in the presence of 50 mM KCl, 10 mM potassium phosphate buffer, 0.6 mM DNA concentration per strand, pH 7.0 and 25 °C on a 600 MHz NMR spectrometer. Signals of 12 imino protons, which indicate the formation of three G-quartets, are marked with squares. (**b**) Detailed comparison of aromatic and methyl regions of 1D ^1^H NMR spectra of RAN1 and RAN1*. Signals used in evaluation of the population of the unknown species in the sample are marked with an asterisk.

20-nt RANwt is comprised of one G-tract with four guanines and three GGG-tracts. Imino region of 1D ^1^H NMR spectrum of RANwt shows several weak, overlapping signals, indicating formation of multiple G-quadruplex structures in the presence of KCl (Figure 1). Minor sequence modifications in the first G-tract of RANwt, where we individually substituted G1 and G4 with thymines, greatly diminished structural polymorphism and led to single G-quadruplex conformations. While we showed that G4-to-T4 modification (RAN4) results in formation of a two-quartet G-quadruplex further stabilized by A•G•A and G•G•G base triads [40], 1D ^1^H NMR spectrum of oligonucleotide with G1-to-T1 modification, RAN1, indicates formation of a G-quadruplex with three G-quartets, as substantiated by observation of 12 clearly resolved signals between *δ* 10.5 and 12.0 ppm (Figure 1a). CD profile of RAN1 with minimum at 245 nm and maximum at 264 nm suggests parallel orientation of the G-strands within formed G-quadruplex(es) (Figure S1).

Although a single set of 12 signals in the imino region of 1D ^1^H NMR spectrum of RAN1 suggests formation of a single G-quadruplex, detailed analysis of aromatic and methyl regions revealed the presence of another conformation in the sample (Figure 1b; for the comparison of the full 1D ^1^H NMR spectra of RAN1 and RAN1* see Figure S2). Despite the fact that RAN1 sequence contains only one thymine, we observed two signals in the methyl region of the 1D ^1^H NMR spectrum at around *δ* 1.2 and 1.7 ppm, where the first signal belongs to T1 of the predominant RAN1 G-quadruplex and the latter to the T1 of the minor species. Based on the relative ratio between these two signals we deduced that the additional species represents a population of about 40% (Figure 1b). Consequently, 2D NOESY spectra of RAN1 showed severe overlap in the aromatic-sugar region, impeding spectral assignments, and detailed structural characterization (Figure S3). By introducing additional A17-to-T17 modification to the RAN1 sequence (herein referred as RAN1*) we reduced the population of unknown species to less than 10% (Figure 1 and Figure S3). The reduction was evaluated based on intensities of signals belonging to methyl groups in RAN1*: signal at *δ* 1.7 ppm in 1D ^1^H NMR spectrum decreased significantly upon A17-to-T17 modification in comparison to signals of T1 and T17 methyl groups at *δ* 1.2 and 1.8 ppm, respectively (Figure 1b). Additionally, influence of A17-to-T17 modification on reducing population of unknown species was evaluated by comparison of the aromatic regions of 1D ^1^H NMR spectra of RAN1 and RAN1*. We especially focused on signal at *δ* 7.2 ppm, whose intensity reduced dramatically in RAN1* and was based on the lack of NOE connectivities to the G-quadruplex-forming residues attributed to the unknown species (Figure 1b and Figure S3). A17-to-T17 modification enabled the assignment and structural characterization of RAN1* since it resulted in more clear 2D NOESY spectra with considerably less spectral overlap, while keeping the chemical shifts of signals belonging to predominant G-quadruplex intact (Figure S3). Noteworthy, detailed comparison of NMR and CD spectra of RAN1 and RAN1* demonstrated that A17-to-T17 modification did not perturb G-quadruplex structure, which retained its three G-quartet topology with parallel arrangement of G-rich strands (Figure 1 and Figure S1). Preserved fingerprint of proton resonances in the imino region and NOESY traces in the aromatic-sugar region of RAN1* together with poor dispersion of the majority of the aromatic protons of the unknown species (between *δ* 7.4 and 7.9 ppm) indicates that the latter belongs to the unfolded form of the investigated oligonucleotide.

### 2.2. RAN1* Forms a Parallel G-Quadruplex with a G-A Bulge

**Figure 2:**
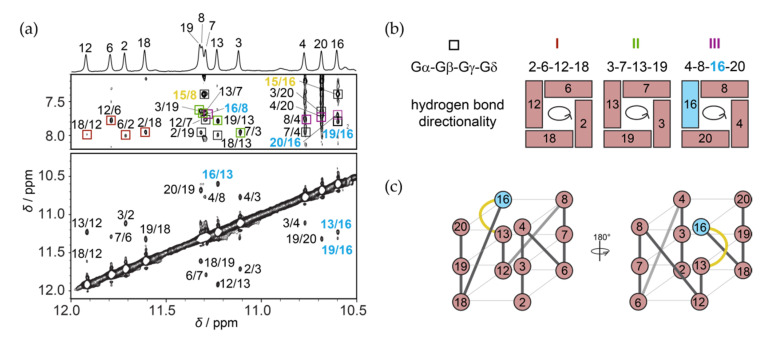
NMR spectral assignments and folding topology of RAN1*. (**a**) Imino region of assigned 1D ^1^H NMR spectrum of RAN1* (top) and imino-aromatic and imino-imino regions of the corresponding 2D NOESY NMR spectrum (mixing time of 300 ms). Imino-H8 cross-peaks labeled with dark red, green, and violet rectangles correspond to intra-quartet connectivities within the G2•G6•G12•G18, G3•G7•G13•G19 and G4•G8•G16•G20 quartets, respectively. Cross-peaks labeled with black rectangles belong to imino-H8 NOE connectivities between neighboring G-quartets. All NOEs involving G16 are marked with light blue, while NOE contacts suggesting stacking of A15 with neighboring G-quartet are highlighted in yellow. (**b**) G-quartets I, II, and III with the corresponding donor-to-acceptor hydrogen bond directionalities. (**c**) Schematic presentations of G-quadruplex adopted by RAN1* with three propeller-type loops (grey) and a bulge (yellow). Residues in G-quartet core adopting *anti* and *syn* glycosidic conformations are colored salmon and light blue, respectively.

Based on the sequence of RAN1* and its NMR spectra (Figure 1) we expected that a three G-quartet core will be composed of 12 guanines from the four continuous GGG-tracts, while connecting residues will form three propeller-type loops in a parallel-stranded G-quadruplex (for detailed description of loop types typically found in G-quadruplex structures see Figure S4). We unambiguously assigned imino protons of RAN1* by performing 1D ^15^N-edited HSQC experiments acquired on residue-specific 6% ^15^N/^13^C-isotopically labeled oligonucleotides (Figure S5). In contrast to expectations, results show that G14 from the third G-tract is not engaged in the formation of a G-quartet and that the signal at *δ* 10.6 ppm belongs to G16 (Figure 2a, top). Inspection of imino-H8 connectivity pattern in the NOESY spectra of RAN1* revealed that G-quartets I, II, and III consist of residues G2•G6•G12•G18, G3•G7•G13•G19 and G4•G8•G16•G20 with anticlockwise hydrogen bond directionalities. In more detail, in the G2•G6 base pair from G-quartet I for example, G2 is the donor of hydrogen bond, while G6 acts as its acceptor. Following the donor-to-acceptor course over individual base pairs within a G-quartet we can confirm anticlockwise hydrogen bond directionalities for all three G-quartets when viewed from the 3′-end (as in Figure 2b). Next to imino-H8 NOEs between G16 and adjacent G-quartet forming G8 and G20 residues, the position of G16 above G13 is supported by inter-quartet imino-imino G13-G16 and G19-G16, and imino-H8 G19-G16 NOEs (Figure 2a). Due to the involvement of G16 in the G-quartet III, residues G14 and A15 form a bulge that connects residues G13 and G16 within a discontinuous G-strand (Figure 2c). Imino-H8 NOE contacts between bulge-forming residue A15 and residues G8 and G16 suggest stacking of A15 on G-quartet III (Figure 2a). On the other hand, lack of NOE contacts between H8 and H1 protons of G14 and neighbouring residues indicate its dynamic nature. NOE connectivities between T1 and G18 on the other side of the G-quadruplex core indicate stacking of T1 to G-quartet I.

The parallel strands of RAN1* G-quadruplex core are linked by three propeller loops that progress anticlockwise and are forming a –(ppp) topology [22,23] (Figure 2c). Although –(ppp) topology is usually characterized by all *anti* glycosidic conformations of G-quartet–forming guanines [22,23], intense intra-residue H8–H1′ NOE cross-peak indicates *syn* glycosidic conformation of G16 (Figure S6). This observation is corroborated by downfield chemical shifts of G16 H2′ and H2′′ protons, which resonate at *δ* 3.70 and 2.73 ppm, respectively, and carbon C8 at *δ_C_* ≈ 140.8 ppm. Moreover, we observed intense NOE cross-peaks between H8 and H1′ of G16 and sugar protons of G13 (especially H1′). Concurrent observation of these connectivities suggest that H8 and H1′ of G16 are both oriented towards sugar moiety of G13, which is only possible if G16 is in *syn* glycosidic conformation (Figure S6).

### 2.3. Solution Structure of the RAN1* G-Quadruplex

**Figure 3:**
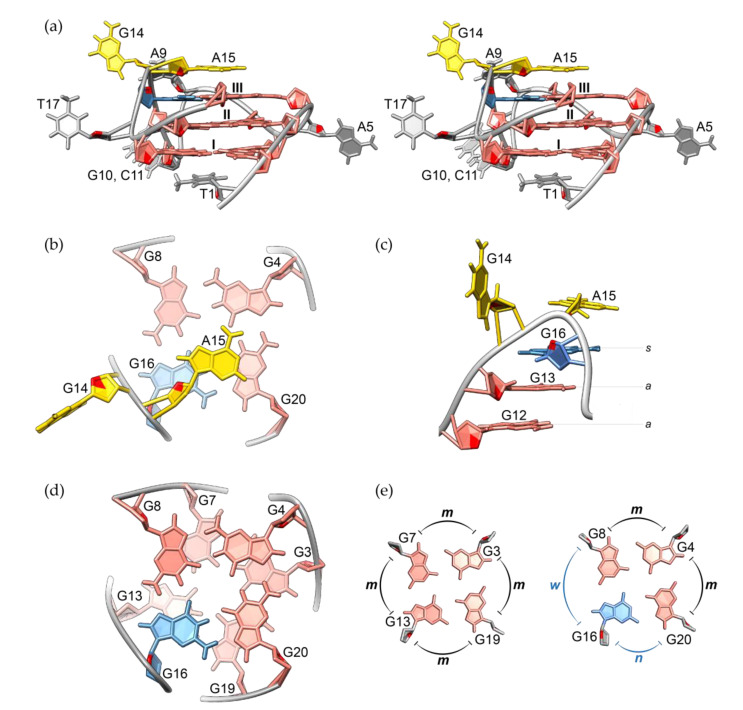
(**a**) Stereo-view of the lowest-energy structure of RAN1*. G-quartets are marked I, II and III. (**b**) Stacking interactions between A15 and G-quartet III. (**c**) Side-view of the G14-A15 bulge and G12-G13-G16 residues forming a discontinuous G-tract. (**d**) Stacking interactions between G-quartets II and III. (**e**) Comparison of groove widths between G-quartets II and III. Letters *m*, *w*, and *n* denote medium, wide and narrow grooves, respectively. Residues with *anti* and *syn* glycosidic conformations are colored salmon and light blue, respectively. Bulge residues G14 and A15 are in yellow, while other loop residues are grey. O4′ atoms are red.

High-resolution structure of RAN1* revealed unprecedented structural details of the G-quadruplex with a G-A bulge. Structural calculations were conducted based on 630 NOE-derived distance restraints, along with 24 hydrogen bond, 37 torsion angle, and 36 planarity restraints using restrained simulated annealing protocol (Table 1). Results show that RAN1* folds into a parallel G-quadruplex with three G-quartets connected by two single-nucleotide (A5 and T17) and one three-nucleotide (A9-G10-C11) propeller loops, and a bulge (G14-A15) (Figure 3a). While both single-nucleotide loops are well defined, A9-G10-C11 segment represents the most dynamic part of the molecule (for ensemble of the ten lowest-energy structures see Figure S7). G2•G6•G12•G18 quartet is stacked by residue T1 from the 5′-end of the RAN1* sequence, while A15 from the bulge stacks on G-quartet III (formed by G4•G8•G16•G20) (Figure 3a,b and Figure S7). In contrast to well defined orientation of A15, G14 is more dynamic and exposed to the solvent (Figure S7). Overall pairwise heavy atom RMSD value of the ten lowest-energy structures (1.55 Å) reflects the dynamic nature of residues A9-G10-C11 from the middle propeller loop and G14. Consequently, RMSD excluding these residues from the calculation is significantly lower (0.51 Å). Comparable RMSD value of 0.41 Å calculated for G-quartets only (i.e., excluding residues T1, A5, A15, and T17) indicates that well-defined loop residues and residues that stack on the G-quartets tightly pack around the core of the RAN1* structure.

One of the most distinctive structural elements of RAN1* G-quadruplex is a G14-A15 bulge, which arises as a consequence of G16 being involved in G-quartet III. Engagement of G16 in a G-quartet leads to a formation of a discontinuous G12-G13-G16 G-tract, where G12 and G13 occupy *anti* glycosidic conformation, while G16 is in *syn* conformation (Figure 3c). In order to properly position G16 over G13 the backbone has to make a turn, where bulge residues G14-A15 act like *pseudo-loop* that connects residues with distinct glycosidic bond angles. Their *pseudo-loop* conformation is especially important at providing the optimized stacking between G-quartets in RAN1*. Despite unique structural features of RAN1*, such as occurrence of two-nucleotide G-A bulge and *syn* glycosidic conformation of G16, the base stacking geometry is, aside from a minimal shift, not significantly affected (Figure 3d). Sequential stacking of guanines in RAN1* follows a so-called partial 5/6-ring stacking geometry, which is typical for structures with parallel orientations of G-strands, where intra-strand guanines progress in *anti*-*anti* base-steps [19,22,43]. Although G13 and G16 represent an *anti*-*syn* base-step, bulge residues G14-A15 compensate the change in glycosidic bond angle and allow for proper partial 5/6-ring stacking. Minimal discrepancy from the regular partial 5/6-ring stacking, where 5-member ring of one guanine partially overlaps with 6-member ring of the other, may be attributed to changes in groove widths arising as a consequence of *syn* glycosidic conformation of G16. G-quartets I and II consist of residues that all occupy *anti* glycosidic torsion angles. Hence, all four grooves defining this part of G-quadruplex core are of medium size with average P-P distance of ~16.3 Å (Figure 3e, Table S2; for more elaborated display of groove widths see Figure S8). In contrast to G-quartets I and II, G-quartet III is characterized by *anti*-*anti*-*syn*-*anti* arrangement of glycosidic conformations. Consequently, G-tracts around G-quartet III form one narrow (P-P distance 9.3 Å), one wide (P-P distance 19.4 Å) and two medium grooves (P-P distances 16.3 and 16.1 Å, Figure S8, Table S2). Differences in groove widths cause minor shift in stacking geometry at G7-G8 and G13-G16 base-steps, while G3-G4 and G19-G20 are less affected. The effect is thus limited to the side of the structure where the groove changes from medium to wide and the distance between phosphate groups is greater by approximately 2 Å (17.3 Å as a medium groove that widens to 19.4 Å). Although the difference in P-P distance is even bigger at the side of narrow groove formation, where the groove dimension changes by more than 6.5 Å, the change in base stacking is not so pronounced.

### 2.4. Effect of a Bulge Modifications on the RAN1* Structure

**Figure 4:**
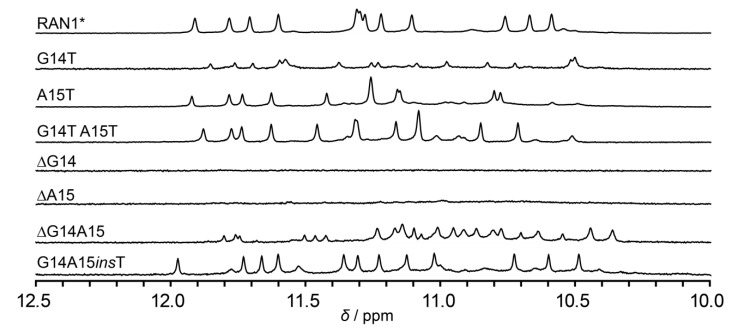
Imino regions of RAN1* and its modified sequences.

Inspired by unprecedented structural features of RAN1*, we prepared a set of constructs bearing different modifications of bulge-forming G14 and A15 with the goal to explore the role of these residues in the formation and integrity of RAN1* G-quadruplex (Figure 4). Modifications included replacement of G14 and/or A15 to thymines and individual or double deletion of bulge-forming residues (Table S1; note: all sequences used in 2.4. are derived from RAN1* sequence, however, the names of constructs are shortened for clarity, for example G14T denotes RAN1* G14T). The individual or double replacements of G14 and A15 to thymines did not disrupt the three G-quartet core, as indicated by 12 signals in the imino regions of the respective 1D ^1^H NMR spectra (Figure 4). Different chemical shifts of imino protons of A15T and G14T A15T in comparison to RAN1* are probably a consequence of altered stacking between thymine at position 15 and G-quartet III upon modification. Interestingly, G14T modification leads to reduction of G-quadruplex formation by 80% thus leaving most of the oligonucleotide unfolded, although G14 is not engaged into any G-quartet nor it forms any NMR-detectable interaction with other residues. Individual deletions of G14 or A15 impede formation of RAN1* G-quadruplex (or any other secondary structure stabilized by base pairing involving imino protons) as indicated by lack of signals in the imino regions of 1D ^1^H NMR spectra of ∆G14 and ∆A15 constructs.

On the other hand, more than 20 signals in the imino region of the 1D ^1^H NMR spectrum of ∆G14A15 construct indicates formation of multiple species in a modified RAN1* with double deletion. Although ∆G14A15, based on its sequence 5′-d(TGGGAGGGAGCGGGTGGG)-3′ alone, allows for formation of the G-quadruplex core similar to RAN1* (three-layered core, but without a bulge), no signals characteristic for RAN1* were observed. This indicates that ∆G14A15 folds into G-quadruplex structures distinct from topology of the parent RAN1*. Moreover, the detrimental effects of G14 and/or A15 deletions on the RAN1* G-quadruplex structure suggest that *pseudo-loop* arrangement can only connect G13 and G16 in *syn* glycosidic conformation if the bulge is at least two residues long. Intrigued by this observation, we designed a construct with inserted thymine residue between G14 and A15 to test the ability of a G-quadruplex formation with a longer bulge. Similar distribution of 12 signals in the imino region of 1D ^1^H NMR spectrum of G14A15*ins*T in comparison to RAN1* corroborates the formation of a G-quadruplex with prolonged G-T-A bulge.

## 3. Discussion

20 nt long G-rich sequence found in the regulatory region of the *RANKL* gene, RANwt, encompasses three G-tracts with three guanines and one G-tract with four guanine residues. Four successive guanines in the first G-tract of the sequence (G1 to G4) are most probably the cause of severe structural polymorphism of RANwt (Figure 1). Introduction of simple G1-to-T1 and G4-to-T4 modifications result in RAN1 and RAN4 samples, respectively, that have been shown to fold into a single G-quadruplex conformation. We recently described a RAN4 G-quadruplex structure with two G-quartets stabilized by A•G•A and G•G•G base triads, which emerges after residue G4 from the first G-tract is replaced by a thymine [40]. Examination of the sequence bearing a G1-to-T1 modification, which was performed in this study, revealed that it folds into a G-quadruplex structure fundamentally different from the one observed for RAN4. A sample suitable for structural characterization was gained by additional A17-to-T17 modification in RAN1*. NMR and CD spectral fingerprints of RAN1 and RAN1* clearly show that introduction of additional A17-to-T17 modification does not influence the G-quadruplex fold, while it significantly reduces the amount of unfolded species in the sample (Figure 1, Figures S1–S3).

The high-resolution structure of RAN1* uncovers a parallel-stranded G-quadruplex topology with three G-quartets. However, in contrast to expectations, guanines engaged in G-quartets are not all originating from G_3_-tracts; one of the residues comprising G-quartet III is G16 from A15-G16-T17 section connecting the third and fourth G-tracts (Figure 2). Due to incorporation of G16 into the G-quadruplex core, residues G14 and A15 are pushed into a bulge resulting in formation of discontinuous G12-G13-G16 G-tract. Since G16 occupies *syn* glycosidic conformation, G14 and A15 arrange in a so-called *pseudo-loop*, which facilitates stacking of G13 and G16 and formation of parallel-stranded topology. The positions of G14 and A15 are reminiscent of a lateral loop that connects guanines with different glycosidic bond angles from two antiparallel G-strands [22,23]. To accommodate antiparallel orientation of G-strands connected by lateral loop, the loop has to be at least two nucleotides long [44,45]. The replacements of G14 and/or A15 for thymines in RAN1* are well tolerated, while the structure is particularly sensitive to their individual deletions. Similarly to the strong influence of the number of the nucleotides needed for the lateral loop formation, our results show that *pseudo-loop* has to be at least two residues long to uphold demanding conformational limitations of the backbone at the position of the bulge (Figure 4).

**Figure 5:**
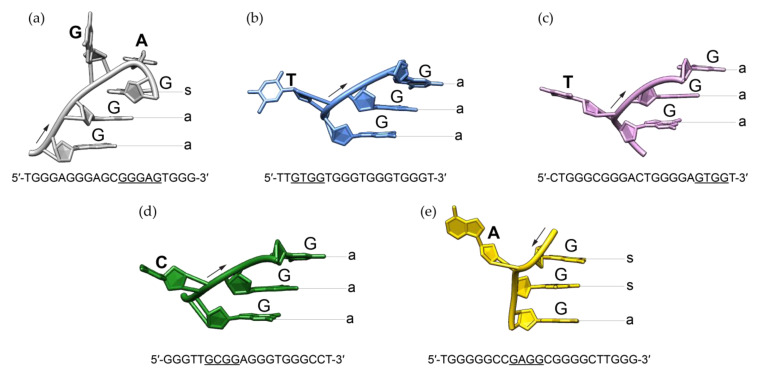
Comparison of bulge-containing G-strand progression between (**a**) RAN1* (this work, PDB ID 6ZRM), (**b**) *TB-1* (PDB ID 2M4P) [27], (**c**) *LTR-IV* (PDB ID 2N4Y) [28], (**d**) *hTR 1-20* (PDB ID 5UA3) [29] and (**e**) *TP3* (PDB ID 6AC7) [30]. G-tract that includes a bulge and whose structure is presented is underlined in a sequence below individual figure. While sequences from (**a**–**d**) fold into a parallel-stranded G-quadruplex, (**e**) is characterized by a (3 + 1) hybrid folding topology. *Anti* and *syn* glycosidic conformations are marked with *a* and *s*, respectively. The arrows indicate the 5′-to-3′ progression of the backbone.

Detailed comparison of RAN1* G-quadruplex to previously described structures containing bulges revealed significant structural differences including the progression of the backbone and related glycosidic conformations of residues next to the bulge (Figure 5). In the last decade, structures bearing single-nucleotide thymine [27,28], cytosine [29], and adenine [30] bulges were observed. Intramolecular G-quadruplex structures with T-bulges formed by G-rich sequences *TB-1* and *LTR-IV* from the HIV-1 virus adopt a parallel topology [27,28]. High resolution X-ray structure of a G-quadruplex with C-bulge revealed the formation of intramolecular parallel-stranded units that stack via 5′-quartets [29]. Additionally, recently published structural study showed that G-rich sequence from *PARP1* promoter forms a three-layered intramolecular (3 + 1) hybrid G-quadruplex scaffold, which exhibits an A-bulge [30]. Regardless of the type of the protruding residue or the folding topology these structures adopt, bulge is in all cases connecting two guanines with the same glycosidic bond angle, i.e., two guanines with *anti* glycosidic conformations if G-strands are oriented parallel to each other [27,28,29] or two *syn* guanines in *PARP1* (3 + 1) hybrid topology [30]. In contrast to all other *anti* guanines from the G-quadruplex core of RAN1*, G16 occupies *syn* glycosidic torsion angle. G-A bulge in the G-quadruplex structure of RAN1* thus connects two guanines with different glycosidic conformations. This significantly influences the progression of the backbone of the bulge-containing G-strand (Figure 5a). In order to be engaged in the G-quartet III, the base moiety of G16 has to be orientated in a way that enables intact hydrogen bond formation with guanines from the neighboring G-strands. At the same time, it has to be positioned above G13 to ensure partial 5/6-ring stacking interactions, typical for parallel G-quadruplex structures [19,22,43]. Apparently, the required *syn* glycosidic conformation of G16 can only be achieved by a backbone turn at the position of bulge by forming a *pseudo-loop*. In contrast to G14-A15 *pseudo-loop* in the G-quadruplex adopted by RAN1*, bulges of other structures are protruding away from the G-quadruplex core without significantly influencing the progression of the backbone (Figure 5). Structural analysis suggests that *syn* glycosidic conformation of G16 influences the groove widths that embrace the G-quadruplex core of RAN1*. We observed a change from four medium grooves to one wide, one narrow, and two medium. On the other hand, groove widths in other bulge-containing structures are consistent with their folding topology: parallel-stranded *TB-1*, *LTR-IV*, and *hTR 1-20* exhibit four medium grooves, while *TP3* (3 + 1) hybrid structure is defined by one narrow, one wide, and two medium grooves [27,28,29,30].

Characterization of parallel-stranded RAN1* structure with a G-A bulge as one of the possible G-quadruplexes that are able to form by a G-rich sequence from regulatory region of *RANKL* gene raises several exciting points. First of all, RAN1* structure together with antiparallel-stranded RAN4 [40] represents topologically distinct pair of non-canonical structures that may differently influence the expression of a *RANKL* gene. Furthermore, special structural features of RAN1*, such as *pseudo-loop*, can represent unique platform for binding to protein partners. Interestingly, some of the G-quadruplex-binding proteins preferentially recognize the phosphate backbone of the loop, regardless of the exact G-quadruplex topology or even DNA sequence or simultaneously interact with G-quartet and loop [46,47]. On the other hand, prominent structural elements protruding from the G-quadruplex core can represent an obstacle for effective topology-specific helicase recognition [48,49,50]. Decreased function of a resolvase may thus affect G-quadruplex unwinding and significantly alter the expression of a gene [48,50,51].

Structural details of G-quadruplex adopted by RAN1* presented in this study offer unique opportunity to anticipate and predict possible structural motifs, which could be used to stabilize the desired G-quadruplex structure. Due to conformational similarity between different G-quadruplexes, the high target selectivity is hard to achieve and the design of a small-molecule ligand that could discriminate a single G-quadruplex target is remarkably challenging [4,51,52,53]. Structural features, not shared by many G-quadruplexes, such as *pseudo-loops*, can provide a rationale for development of specific ligands, which would allow for explicit preference for one G-quadruplex over another. Stacking of A15 on G-quartet III and dynamic residues A9 and G14 can hinder the access and stacking of the potential ligand onto the 3′-G-quartet (Figure 3 and Figure S7). Although steric restrictions can prevent the effective binding of a ligand to one of the G-quartets, they can as well be used as a tool with which we direct the ligand to other high-affinity binding sites on the other side of the G-quadruplex core [54]. Next to unique loops, targeting the grooves represents one of the most promising binding modes to enhance the selectivity of G-quadruplex ligands [55,56,57]. Alterations in groove widths along the progression from one G-quartet to another in RAN1* can serve as a unique platform to design a new generation of highly specific groove binders. Selective binding with the highest effectivity that combines several interaction sites including loops and grooves would demand development of a non-planar ligand with multiple arms, such as platinum(II)-based tripod (Pt-tripod) [58]. Not only that the Pt-tripod stacks to the 5′-G-quartet of human Tel26 G-quadruplex and stretches two of its arms into the grooves, the ligand induces a formation of hydrogen-bonded base triad, additionally enhancing its tight and specific binding [58].

Last but not least, unprecedented high-resolution structure of RAN1* G-quadruplex and detailed description of *pseudo-loop* characteristics deepens our knowledge of diversity and complexity of structures that can be anticipated to fold from G-rich sequences. The NMR derived RAN1* structure is, to the best of our knowledge, the first high-resolution structure of a G-quadruplex defined by unprecedented two-nucleotide bulge, in which guanine is followed by an adenine. However, whether the formation of G-quadruplex(es) in the regulatory region of the *RANKL* gene can be correlated with its lower expression is yet to be determined.

Note added in proof: During the revision process of our work, an article related to the topic of unusual G-quadruplex structures was published by Ngoc Nguyen *et al.* in Nucleic Acids Research [59]. The authors determined the structure of a G-quadruplex containing a 15-nt bulge, in which they incorporated a duplex stem. In contrast to a non-structured bulge, increasing the duplex bulge size to 25- and 33-nt resulted in even slightly higher thermodynamic stability. The structure with duplex bulge expands the knowledge of sequences able to form G-quadruplexes and highlights the potential roles bulges might play at designing unique ligands for specific G-quadruplex targeting in the future.

## 4. Materials and Methods

### 4.1. Sample Preparation

All isotopically unlabeled and residue-specific ^15^N/^13^C-labeled RAN1 and RAN1* oligonucleotides were synthesized on K&A Laborgeraete GbR DNA/RNA Synthesizer H-8 using standard phosphoramidite chemistry in DMT-on mode. Oligonucleotides were cleaved from the solid support by treatment with concentrated aqueous ammonia at 55 °C overnight and purified using reverse-phase HPLC chromatography, followed by removal of DMT group with reaction in 80% AcOH for 30 min. Oligonucleotides were then extracted with pure ethanol and 8 M LiCl. Samples were after rinsing with 70% ethanol dissolved in pure water. The pH value of samples was adjusted to around 7 using LiOH. Samples were subsequently desalted using ultrafiltration cell with 1 kDa cellulose membrane. Concentration of desalted samples was determined by UV absorption at 260 nm using UV/VIS Spectrophotometer Varian CARY-100 BIO UV–VIS. Extinction coefficients were determined by the nearest neighbor method. 10% of ^2^H_2_O, 50 mM KCl, and 10mM potassium phosphate buffer with pH 7.0 were added to the samples. The pH of the oligonucleotide samples was adjusted to 7.0 using HCl or LiOH. The samples were heated up to 90 °C for 5 min and then slowly cooled down to room temperature.

### 4.2. CD and UV Experiments

Circular dichroism (CD) spectra were recorded on an Applied Photophysics Chirascan CD spectrometer at 25 °C using a quartz cuvette with a 1.0 mm path length. The wavelength range was from 220 to 320 nm. CD samples were prepared at 30 μM oligonucleotide concentrations in 50 mM KCl and 10 mM potassium phosphate buffer. A blank sample containing only 50 mM KCl and 10 mM potassium phosphate buffer was used for baseline correction. Three scans were averaged to obtain each CD spectrum.

UV melting experiments of RAN1 and RAN1* were performed on a Varian CARY-100 BIO UV–VIS spectrophotometer with the Cary Win UV Thermal program using a 1.0 cm pathlength cells. Samples for UV measurements were prepared at 30 μM oligonucleotide concentrations in 50 mM KCl and 10 mM potassium phosphate buffer. Samples were heated at 0.1 °C min^−1^ from 10 °C to 90 °C and absorbance at 295 nm was measured at 0.5 °C steps. Mineral oil was used to prevent evaporation and sample loss due to high temperatures. A stream of nitrogen was applied throughout the measurements to prevent condensation at lower temperatures. T_m_ was determined from the normalized A_295_ versus temperature plot.

### 4.3. NMR Experiments and Structure Calculations

NMR experiments were recorded on Agilent-Varian VNMRS 600 MHz and 800 MHz spectrometers at 25 °C. Double-pulsed field gradient spin echo (DPFGSE) pulse sequence was used to suppress the water signal. Identification of guanine imino protons in residue-specific 6% ^15^N, ^13^C-labeled samples of RAN1* was performed by 1D ^15^N-edited HSQC NMR experiments. Exchangeable and non-exchangeable proton resonances of RAN1* were assigned using 2D NOESY spectra (mixing times of 50, 100, 200, 300, and 400 ms) recorded in 90% H_2_O/10% ^2^H_2_O. The 2D DQF-COSY and TOCSY (mixing time of 40 ms) spectra were used to estimate sugar conformations. All spectra were processed in VNMRJ program (Agilent Technologies). Resonance assignment and integration were achieved using SPARKY software [60]. NOE distance restraints for non-exchangeable protons were obtained from 2D NOESY spectrum recorded at 25 °C with a mixing time of 300 ms. Only non-overlapping cross-peaks were integrated and used for distance restraints calculation. An average volume of intra-residual H8-H1′ NOE correlation was used as the reference volume. Because residues G2, G3, G4, G6, G7, G12, G13, G18, and G20 did not overlap with any other cross-peak and clearly exhibited *anti* glycosidic conformations, we could reference the averaged volume to the value of 3.9 Å. With the help of this reference, we classified the remaining signals as strong (1.8–3.6 Å), medium (2.6–5.0 Å), and weak (3.5–6.5 Å).

NOE distance restraints for exchangeable protons were obtained from 2D NOESY spectrum recorded at 25 °C with a mixing time of 300 ms. Cross-peaks were classified as medium (2.6–5.0 Å) or weak (3.5–6.5 Å) based on their intensities. Torsion angle χ along glycosidic bond was restrained to a range between 200 and 280° for *anti* and between 25 and 95° for *syn* (G16) guanine residues. Torsion angle χ restrains for all adenine residues were set between 200 and 280° and between 170 and 310° for thymine and cytosine residues describing their *anti* glycosidic conformation. Torsion angle ν_2_ was used to restrain sugar pucker to C2′-endo conformation for all residues except G10, G14 and G16, which were left unrestrained.

Structure calculations were performed with AMBER 14 software [61] using parmbsc0 force field [62] with parmχOL4 [63] and parmεζOL1 [64] modifications. The initial extended structure of RAN1* oligonucleotide was obtained with LEAP module of the AMBER 14 program. A total of 100 structures were calculated in 100 ps of NMR restrained simulated annealing (SA) simulations using the generalized Born implicit model to account for solvent effects. The cut-off for non-bonded interactions was 20 Å and the SHAKE algorithm for hydrogen atoms was used with the tolerance of 0.0005 Å. SA calculations were initiated with random velocities. After 5 ps at 300 K, the temperature was raised to 1000 K in the next 10 ps and held constant at 1000 K for 25 ps. Temperature was scaled down to 100 K in the next 35 ps and reduced to 0 K in the last 25 ps. Restraints included in the calculation involved NOE-derived (force constant 50 kcal mol^−1^ A^−2^) and hydrogen bond distance restraints (force constant 50 kcal mol^−1^ A^−2^), torsion angle χ and ν_2_ restraints (force restraints 200 kcal mol^−1^ A^−2^) and planarity restraints (force constant 20 kcal mol^−1^ A^−2^). Planarity restraints were excluded in the last 25 ps of SA. A family of 10 structures was selected based on the lowest energy and subjected to energy minimization with a maximum of 20,000 steps. Figures displaying structures and their details were visualized and prepared with UCSF Chimera software [65]. The coordinates of the RAN1* G-quadruplex have been deposited in the Protein Data Bank with the accession code 6ZRM. Chemical shifts list has been deposited in the Biological Magnetic Resonance Data Bank with the accession code 34529.

## 5. Conclusions

In this study, we determined an unprecedented three-layered G-quadruplex structure with a G-A bulge. Bulge-forming residues G14 and A15 of a 20 nt G-rich sequence found in the regulatory region of an osteoporosis-related *RANKL* gene arrange in a *pseudo-loop* to allow proper partial 5/6-ring stacking of G-quartets and maintain parallel orientation of G-tracts. Unique structural features, such as *pseudo-loop* and alterations of groove widths along the G-core progression, may serve as targets for highly selective structure-based drug design. Structural insights presented in this study may, in the future, represent the basis for the development of novel therapeutic approaches against osteoporosis. However, the biggest question still remains the correlation between G-quadruplex(es) formation and expression levels of *RANKL* gene.

## Figures and Tables

**Table 1:** NMR and structure statistics.

NMR Statistics	
NOE-derived Distance Restraints	
Total	630
Intra-residual	426
Inter-residual	204
Sequential	103
Long-range	101
Hydrogen bond restraints	24
Torsion angle restraints	37
Planarity restraints	36
Structure Statistics	
Violations	
Mean NOE restraint violation (Å)	0.058 ± 0.016
Max. NOE restraint violation (Å)	0.084
Max. torsion angle restraint violation (°)	2.50
Deviations from idealized geometry	
Bonds (Å)	0.0121 ± 0.0002
Angles (°)	2.33 ± 0.03
Pairwise Heavy Atom RMSD (Å)	
Overall	1.55 ± 0.49
Overall without A9-G10-C11 and G14	0.51 ± 0.18
G-quartets	0.41 ± 0.17

## Supplementary Materials

The following are available online, Figure S1: CD spectra and UV melting temperature experiments of RAN1 and RAN1* constructs; Figure S2: Full 1D ^1^H NMR spectra of RAN1 and RAN1*; Figure S3: The aromatic-sugar H2′/H2′’ regions of NOESY spectrum of RAN1 and RAN1*; Figure S4: Schematic presentation of a G-quadruplex topology with all three possible loop types: propeller, diagonal, and lateral (sometimes also referred as edge-wise). Figure S5: Unambiguous assignment of imino proton resonances of RAN1*; Figure S6: NMR observables that indicate *syn* glycosidic conformation of G16; Figure S7: Ten lowest-energy structures of RAN1*; Figure S8: Groove dimensions (Å) in RAN1*. Table S1: List of modified RAN1* constructs used in this study; Table S2: Change in groove dimensions (Å) of RAN1* at the site of G16 in *syn* glycosidic conformation.
