# Supplementary material for: Structure of a DNA G-Quadruplex Related to Osteoporosis with a G-A Bulge Forming a Pseudo-loop
Source: Molecules. 2020 Oct 21;25(20):4867. doi: 10.3390/molecules25204867 (PMC7588008; doi:10.3390/molecules25204867)
Supplement: Supplementary file 1 [file molecules-25-04867-s001.pdf]

Supplementary Information

Structure of a DNA G-quadruplex related to osteoporosis with a G-A bulge forming a *pseudo-loop*

Martina Lenarčič Živković [1,2,*], Jan Rozman [1] and Janez Plavec [1,3,4,*]

[1] Slovenian NMR Centre, National Institute of Chemistry, Hajdrihova 19, 1000 Ljubljana, Slovenia; jan.rozman@gmail.com

[2] Central European Institute of Technology, Masaryk University, Kamenice 753/5, 60177 Brno, Czech Republic

[3] EN-FIST Centre of Excellence, Trg OF 13, Ljubljana 1000, Slovenia

[4] Faculty of Chemistry and Chemical Technology, University of Ljubljana, Večna pot 113, 1000 Ljubljana, Slovenia

* Correspondence: janez.plavec@ki.si (J.P.); martina.lenarcic@ki.si (M.L.Ž.)

Table S1. List of modified RAN1* constructs used in this study.

Name of the Construct	Sequence (5′ → 3′)	Lenght of the Construct (nt)
G14T	TGGGAGGGAGCGG**T**AGTGGG	20
A15T	TGGGAGGGAGCGGG**T**GTGGG	20
G14TA15T	TGGGAGGGAGCGG**TT**GTGGG	20
ΔG14	TGGGAGGGAGCGG**-**AGTGGG	19
ΔA15	TGGGAGGGAGCGGG**-**GTGGG	19
ΔG14A15	TGGGAGGGAGCGG**--**GTGGG	18
G14A15*ins*T	TGGGAGGGAGCGGG**T**AGTGGG	21

Table S2. Change in groove dimensions (Å) of RAN1* at the site of G16 in *syn* glycosidic conformation.

Groove Number	Defined by	Groove Type	Groove Dimension (Å)*	Average Groove Dimension (Å)**	Change in Groove Width (Å)
1	G2-G6	*m*	14.8	16.2	
	G3-G7	*m*	17.5		
	G4-G8	*m*	16.3		
2	G6-G12	*m*	17.3	17.3	
	G7-G13	*m*	17.3		
	G8-G16	*w*	19.4		2.1
3	G12-G18	*m*	14.7	15.9	
	G13-G19	*m*	17.1		
	G16-G20	*n*	9.3		6.6
4	G18-G2	*m*	15.1	16.0	
	G19-G3	*m*	16.8		
	G20-G4	*m*	16.1		

*Groove dimension corresponds to a P-P distance between pairs of guanines from different G-quartets that define the individual groove (Figure S7). Distances were measured in UCSF Chimera Software (Pettersen *et al.*, *J. Comput. Chem.* **2004**, *25* (13), 1605-1612).

**Groove dimension was averaged over the same groove type only.

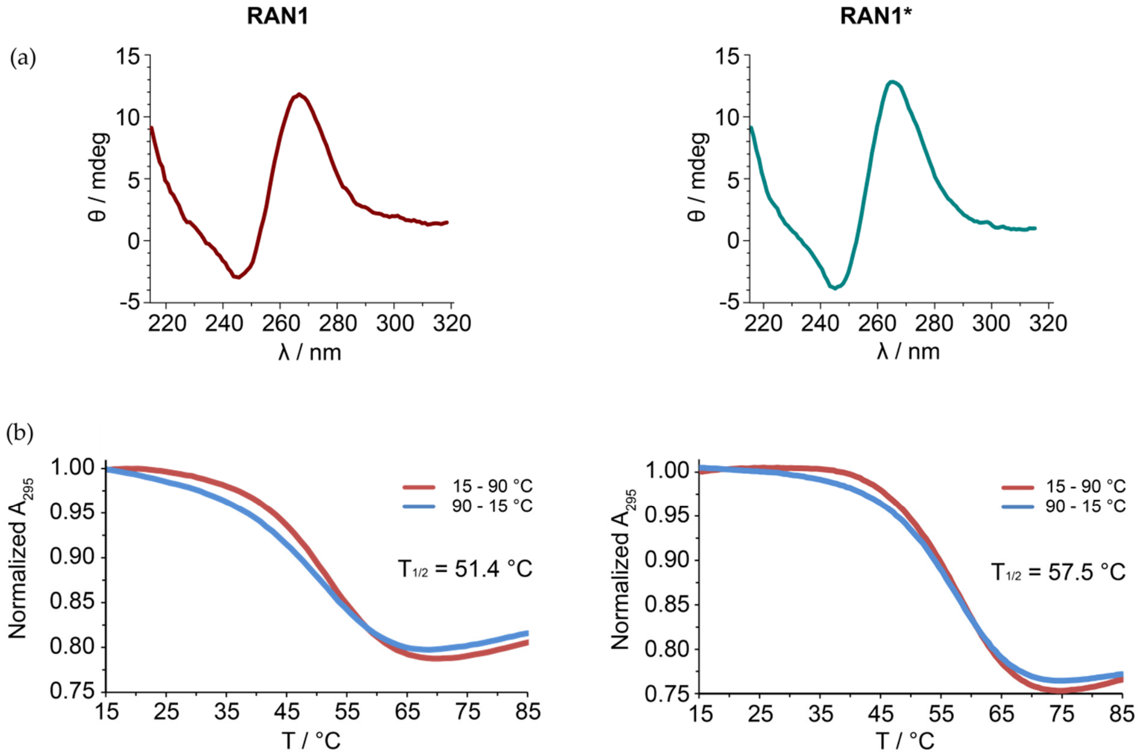

Figure S1. (**a**) CD spectra and (**b**) UV melting temperature experiments of RAN1 and RAN1* constructs.

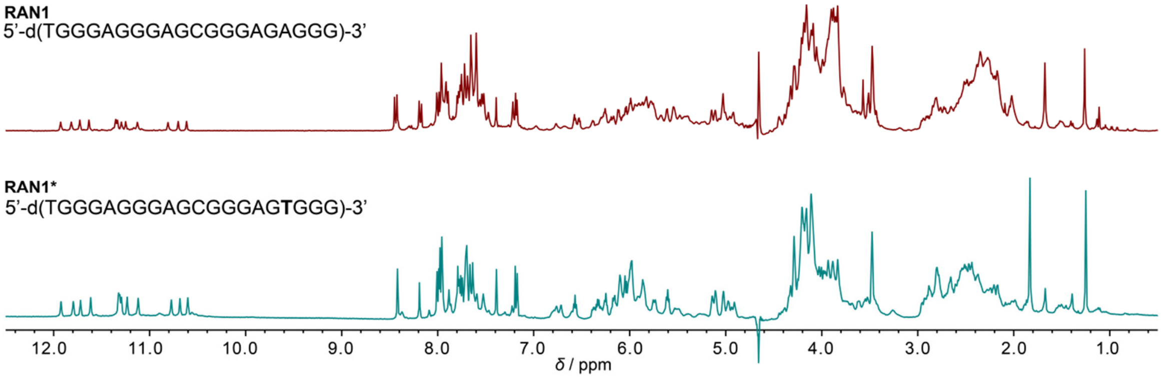

Figure S2. Full 1D ^{1}H-NMR spectra of RAN1 and RAN1* in the presence of presence of 50 mM KCl, 10 mM potassium phosphate buffer, 0.6 mM DNA concentration per strand, pH 7.0 and 25 °C on a 600 MHz NMR spectrometer.

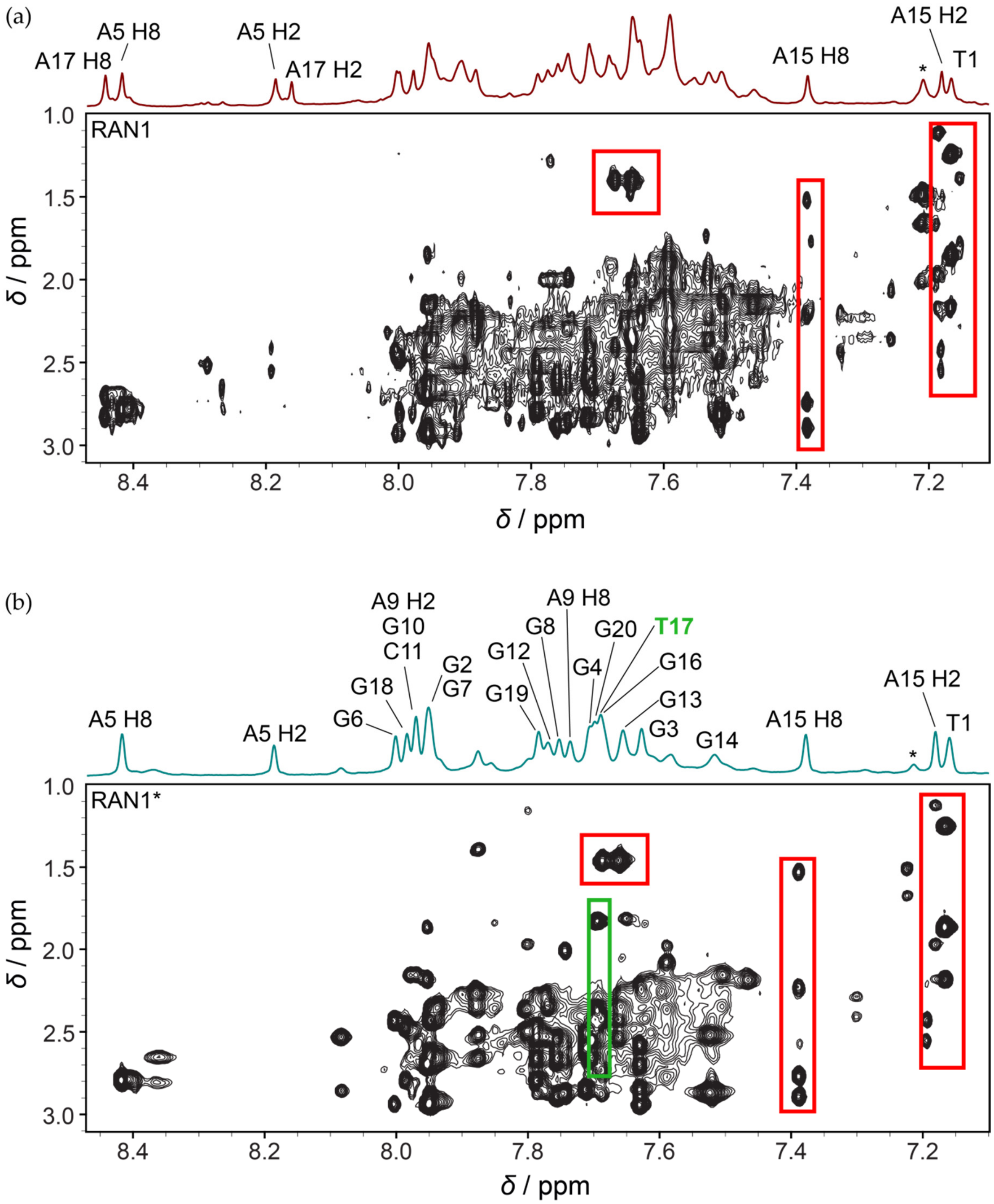

Figure S3. The aromatic-sugar H2′/H2″ region of NOESY spectrum (τ_m = 200 ms) of RAN1 (**A**) and RAN1* (**B**). Representative signal of unfolded species is marked with *. NOE signals distictive of RAN1* structure are boxed with red. Signals of T17 that appear after the A17-to-T17 modification are boxed with green.

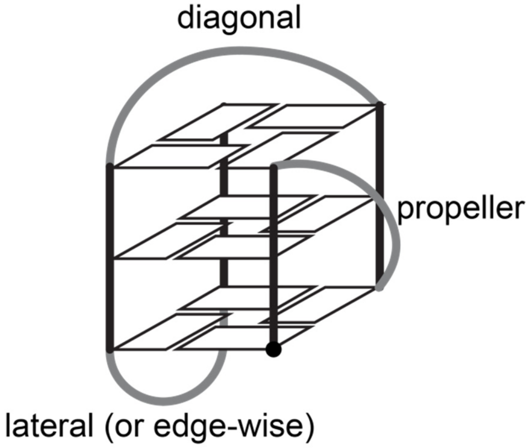

Figure S4. Schematic presentation of a G-quadruplex topology with all three possible loop types: propeller, diagonal, and lateral (sometimes also referred as edge-wise). Propeller-type loop connects two guanines from the top and the bottom G-quartet from adjacent G-strands in parallel orientation. Diagonal loop joins opposite anti-parallel G-strands by connecting two guanines from the same G-quartet. Similarly, lateral loop connects two guanines from the same G-quartet, however, linking adjacent anti-parallel G-strands. Guanines are shown as white rectangles, while loops are coloured grey. The 5′-guanine is marked by a black dot.

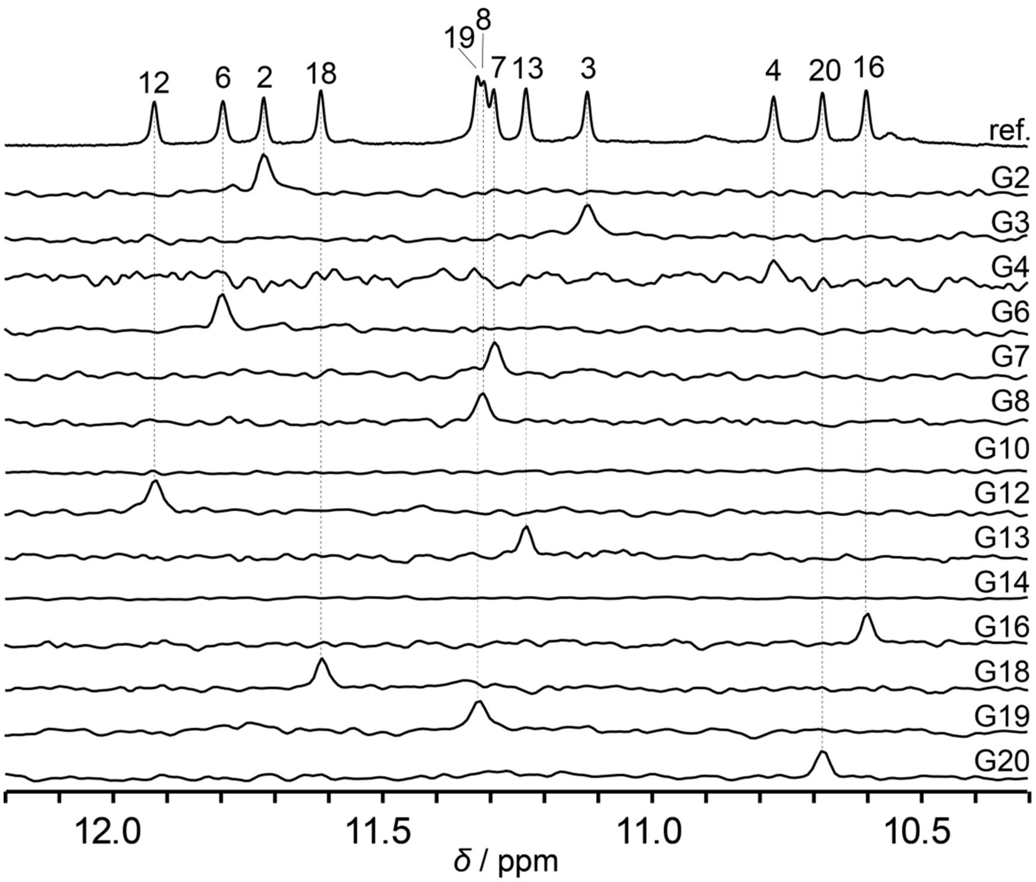

Figure S5. Unambiguous assignment of imino proton resonances of RAN1* was achieved by recording 1D ^{15}N-edited HSQC spectra on partially 6% residue-specific $^{15}N/^{13}C$-labeled oligonucleotides of RAN1*. Imino region of 1D ^{1}H-NMR spectrum of RAN1* (ref.) and assignment of imino resonances are shown on top. Spectra were recorded on Agilent DD2 600 MHz spectrometer at 25 °C in 90% H_2O, 10% $^{2}H_2O$, 50 mM KCl, 10 mM potassium phosphate buffer with pH 7.0. Oligonucleotide concentrations were 0.6 mM.

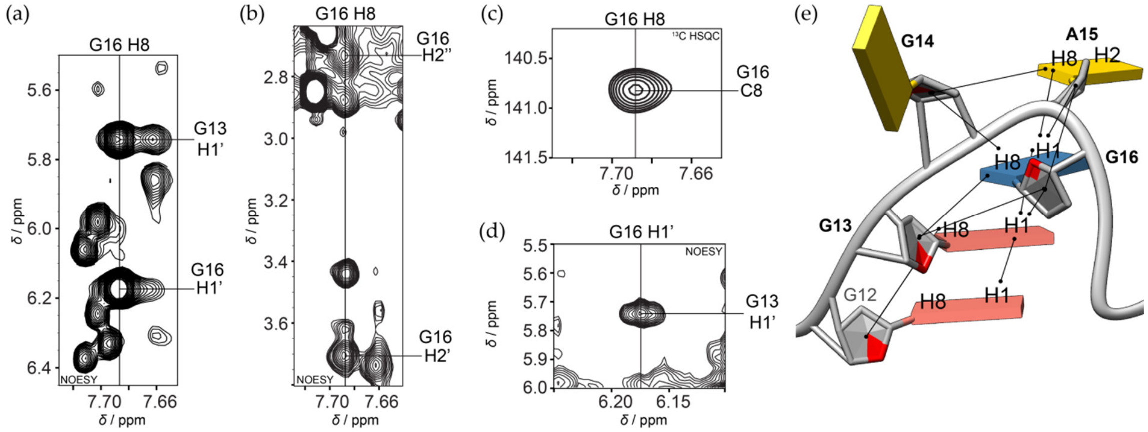

Figure S6. NMR observables that indicate *syn* glycosidic conformation of G16. (**a**) Section of aromatic-anomeric region of NOESY spectra of RAN1* showing intensive intraresidual H8-H1′ NOE contact of G16 and interresidual H8 G16 – H1′ G13 NOE connectivity, respectively. (**b**) Aromatic-H2′/H2″ trace of G16 in NOESY spectra of RAN1*. Downfield chemical shifts of H2' and H2″ corroborate *syn* glycosidic conformation. (**c**) Aroamtic part of 2D ^{13}C-HSQC NMR spectra showing C8-H8 cross-peak of G16. Chemical shift of carbon C8 over 140 ppm is typical for residues in *syn* glycosidic conformation. (**d**) Intense NOE cross-peak between H1′ atoms of G13 and G16. (e) Schematic representation of NOE contacts between G16 and surrounding residues G13, G14, and A15.

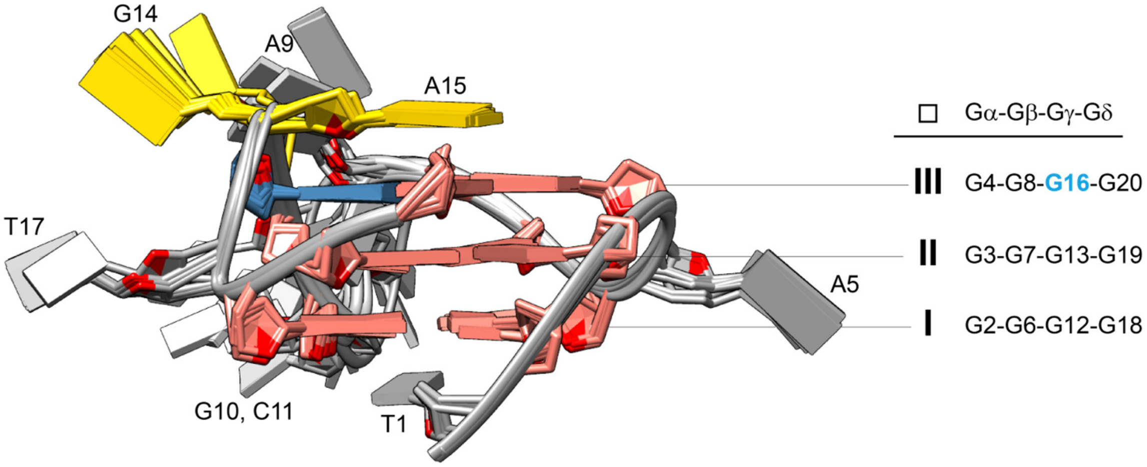

Figure S7. Ten lowest-energy structures of RAN1*. Guanines that participate in G-quartets and are in *anti* glycosidic conformation are colored salmon, while guanine G16 that resides in *syn* confromation is blue. Detailed arrangement of guanines within G-quartets is elaborated on the right. Residues G14 and A15, constituting the bulge, are colored yellow. Other loop residues and backbone are grey. O4′ atoms are red.

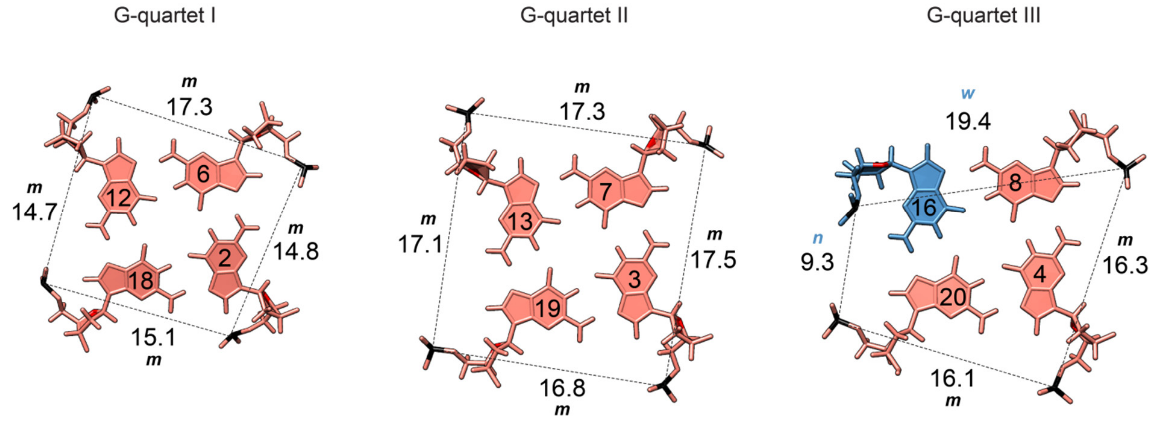

Figure S8. Groove dimensions (Å) in RAN1*. Residues with *anti* and *syn* glycosidic conformations are colored salmon and light blue, respectively. P atoms are colored black. P-P distances between pairs of guanines were measured in UCSF Chimera Software (Pettersen *et al.*, *J. Comput. Chem.* **2004**, 25 (13), 1605-1612) and are depicted with black dashed lines. Labels *m*, *w*, and *n* stand for medium, wide, and narrow grooves, respectively.
